# NRF2: sensing stress, shaping immunity - keeping inflammation quiet and antigens out of sight

**DOI:** 10.3389/fphar.2026.1974746

**Published:** 2026-10-14

**Authors:** Yvonne Benatzy, Bernhard Brüne

**Affiliations:** 1 Institute of Biochemistry I, Faculty of Medicine, Goethe University Frankfurt, Frankfurt, Germany; 2 Frankfurt Cancer Institute, Goethe University Frankfurt, Frankfurt, Germany

**Keywords:** antigen presentation, antioxidant response, *Ciita*, inflammation, MHC class II, NFE2 like BZIP transcription factor 2, nuclear factor erythroid 2-related factor 2

## Abstract

The Cap ‘n’ Collar basic leucine zipper transcription factor NRF2 has been linked to inflammation almost since its identification as the master regulator of oxidative stress responses. Early studies demonstrated systemic inflammation and dysregulated immune responses following NRF2 loss. Initially, NRF2 was thought to regulate pro-inflammatory genes primarily through its antioxidant function, by binding to antioxidant response elements. However, studies by Kobayashi et al. revealed that, in LPS-treated murine macrophages, NRF2 binds to a non-ARE sequence near the *Il6* and *Il1b* genes, where it regulates RNA polymerase II recruitment and represses pro-inflammatory gene transcription. More recent findings have further expanded the role of NRF2 in regulating immune and inflammatory responses by demonstrating that NRF2 suppresses transcription of the MHC class II transactivator (*Ciita*), a master regulator of major histocompatibility complex class II expression and antigen presentation. This article provides an overview of the historical evidence linking NRF2 to inflammatory conditions and discusses the emerging role of NRF2 in the transcriptional control of pro-inflammatory cytokines and *Ciita*. The molecular mechanisms through which NRF2 regulates *Il6*, *Il1b*, and *Ciita* remain incompletely understood and may involve yet-unidentified transcriptional co-regulators, which will be explored in this review.

## 1 Introduction

For nearly 30 years, nuclear factor erythroid 2-related factor (NRF) 2 has been known for its fundamental role in cytoprotection and metabolic regulation through maintaining redox homeostasis and the induction of phase II detoxification enzymes (Itoh et al., 1997; Ishii et al., 2000). Although initially reported to be dispensable for murine development (Chan et al., 1996), homozygous NRF2-deficient mice were subsequently found to be more susceptible to pulmonary injury (Chan and Kan, 1999) and acetaminophen-induced hepatotoxicity as a result of impaired antioxidant response element (ARE)-dependent gene expression (Enomoto et al., 2001). Shortly thereafter, studies of aged female NRF2 knockout (KO) mice broadened the understanding of NRF2 biology. The spontaneous development of a lupus-like autoimmune nephritis in these animals suggested that NRF2 possesses important immunoregulatory functions in addition to its well-established role in antioxidant defense (Yoh et al., 2001). Since then, numerous studies have further established the relevance of NRF2 in inflammatory and immune cell-associated disorders. While some studies attribute the inflammatory and autoimmune phenotypes associated with NRF2 deficiency to impaired antioxidant and detoxification defenses (Li et al., 2004; Yeang et al., 2012; Brüne et al., 2013; Sha et al., 2015; Kuhn et al., 2011), others suggest that NRF2 directly represses genes involved in inflammatory cytokine production (Kobayashi et al., 2016). Recent findings have further delineated NRF2’s immunoregulatory functions.

NRF2 activation has been shown to repress gene and protein expression of co-stimulatory molecules and major histocompatibility complex (MHC) class II molecules, thereby impairing antigen presentation of dendritic cells (DCs) and alveolar macrophages (AMs) along with reducing T cell proliferation (Pham et al., 2026; Mai et al., 2024; Rothchild et al., 2019; Rothe et al., 2015; Wang et al., 2017; Yeang et al., 2012). This review highlights NRF2’s established role in regulating pro-inflammatory gene expression (Ahmed et al., 2017; Battino et al., 2018; Lastres-Becker, 2021; van der Horst et al., 2022) and discusses its so far underappreciated role in regulating MHC class II-mediated antigen presentation. To facilitate consistency and readability, we use a unified nomenclature throughout this review, with protein names capitalized irrespective of species and gene symbols following mouse nomenclature, with only the first letter capitalized.

## 2 NRF2 regulation and its role in inflammation and immunity

### 2.1 Canonical and non-canonical regulation of NRF2 activity

NRF2 protein stability is tightly controlled through the cellular redox state (Itoh et al., 2004). Under basal conditions, NRF2 is constitutively bound to its cytoplasmic binding partner Kelch-like ECH-associated protein (KEAP) 1, which functions as an adaptor of a Cullin (CUL) 3-based E3 ubiquitin ligase complex and thereby targets NRF2 for continuous ubiquitination and proteasomal degradation (Furukawa and Xiong, 2005; McMahon et al., 2003; Zhang et al., 2004). Structural and biochemical studies have shown that NRF2 binding to KEAP1 involves a two-site interaction in which the DLG and ETGE motifs within NRF2’s Neh2 domain engage the Kelch domains of the two KEAP1 subunits with different affinities (Chowdhry et al., 2013; McMahon et al., 2004), resulting in dynamic cycling between single- and dual-site binding configurations (Baird et al., 2013). KEAP1 functions as a homodimer, with the two KEAP1 subunits connected through their bric-a-brac, tramtrack, and broad complex domains. These domains mediate association with CUL3, whereas the C-terminal Kelch domains, also referred to as DC domains, interact with NRF2 (Zhang et al., 2004).

Electrophilic and oxidative stress disrupts this regulatory system by modifying redox-sensitive cysteine residues within KEAP1. In addition to classical reactive oxygen species (ROS), lipid-derived electrophiles, including oxidized polyunsaturated fatty acids (Nagahora et al., 2017) and nitro-fatty acids (Kansanen et al., 2011), can modify KEAP1 cysteine residues. These modifications alter KEAP1 conformation and/or its interaction with the CUL3 ubiquitin ligase complex, thereby reducing NRF2 ubiquitination and allowing NRF2 to accumulate (Suzuki et al., 2025; Kobayashi et al., 2006). KEAP1 contains 25 cysteine residues, several of which have been implicated in sensing distinct electrophilic and oxidative stimuli, primarily Cys273 and Cys288 (Dinkova-Kostova et al., 2002), as well as Cys151 (Suzuki et al., 2025; Zhang and Hannink, 2003). Thus, KEAP1 functions as a redox-sensitive regulatory hub that couples cellular oxidative and electrophilic stress to NRF2 stabilization, enabling subsequent accumulation of NRF2 in the nucleus, where NRF2 can regulate gene transcription. Collectively, this process is commonly known as NRF2 activation.

In addition to the canonical mode of NRF2 regulation, NRF2 signaling can also be regulated through non-canonical mechanisms involving proteins that contain KEAP1-interacting motifs and compete with NRF2 for KEAP1 binding, thereby disrupting the KEAP1-NRF2 interaction (reviewed comprehensively by Silva-Islas and Maldonado (2018)). The autophagy adaptor sequestosome 1 (SQSTM1/p62) is a well-characterized non-canonical regulator of NRF2 that is induced by inflammatory stimuli, including Toll-like receptor activation. It promotes KEAP1 sequestration and autophagy-dependent degradation, thereby enhancing NRF2 stabilization and transcriptional activity (Komatsu et al., 2010; Yin and Cao, 2015).

Binding of KEAP1 by inhibitor of nuclear factor kappa B (NF-κB) kinase subunit beta (IKKβ) represents another mechanism that connects KEAP1-dependent NRF2 regulation with inflammatory signaling (Kim et al., 2010). IKKβ binding to KEAP1 promotes NRF2 stabilization, while simultaneously restraining NF-κB activation, thereby connecting NRF2 regulation to a central transcriptional pathway controlling genes involved in cellular stress responses, proliferation, immunity, and inflammation (Kim et al., 2010). Conversely, NRF2 stabilization promotes its nuclear accumulation and transcriptional activity, driving antioxidant and cytoprotective gene programs and, as discussed in the following sections, modulating inflammatory and immunoregulatory gene expression. Non-canonical regulation thus expands the range of cellular signals controlling NRF2 beyond direct sensing of oxidative or electrophilic stress, linking NRF2 signaling to protein homeostasis, autophagy, and inflammatory pathways. Moreover, it allows NRF2 transcriptional activity to be enhanced in response to cellular stress even in the absence of pronounced oxidative or electrophilic stress (Silva-Islas and Maldonado, 2018; Hayes and Dinkova-Kostova, 2014).

### 2.2 NRF2 is linked to inflammation in mice and humans

Early studies reported that NRF2 KO mice develop a lupus-like autoimmune nephritis (Yoh et al., 2001), a systemic autoimmune disease (Li et al., 2004), and a multiorgan inflammation (Ma et al., 2006). Li et al. (2004) described that NRF2 KO altered the expression of numerous immune-related genes in spleen of female mice. Specifically, the absence of NRF2 upregulated *matrix metalloproteinases 8* and *9*, pro-inflammatory chemokines *C-X-C motif chemokine ligand 10* and *12*, as well as genes that promote B cell differentiation and proliferation. Additionally, NRF2 KO increased expression of *Fc epsilon receptor Ig* (*Fcer1g*, FcεRIγ), which bridges antibodies with cytotoxic cells, allowing antibody-dependent cellular cytotoxicity as well as T cell responses to allergies and tumors (Shao et al., 2026; Zhou et al., 2025). In line with the development of a lupus-like autoimmune disease upon NRF2 loss (Yoh et al., 2001; Li et al., 2004), FcεRIγ expression increased in T cells from patients with systemic lupus erythematosus (SLE) (Enyedy et al., 2001), a complex and systemic autoimmune disease (Tsokos 2011). The strong link between NRF2 and SLE, including NRF2’s involvement and its potential treatment in SLE has been reviewed comprehensively (Ahmad and Ahsan, 2019; Ohl and Tenbrock, 2021; Perl, 2013; Barati and Caster, 2022).

Conversely, NRF2 activation improved lupus nephritis by limiting ROS and by negatively regulating pro-inflammatory pathways (Jiang et al., 2014; Tsai et al., 2011). Moreover, gene expression analysis of CD14^+^ monocytes, isolated from patients with either inactive or active SLE, revealed that monocytes from patients with active SLE showed an increased pro-inflammatory gene expression signature, while in active SLE, NRF2 activity was lower than in inactive SLE monocytes as predicted by ingenuity-pathway-analysis (Labonte et al., 2018). In contrast to pro-inflammatory genes and the later discussed NRF2-dependent repression of major histocompatibility complex (MHC) class II expression found in mycobacteria-infected AMs (Pham et al., 2026), mature DCs (Rothe et al., 2015), and cancer-associated fibroblasts (CAFs) (Zheng et al., 2026), patients with active SLE exhibited a markedly reduced proportion of MHC class II-positive monocytes, which was associated with impaired monocyte-mediated T cell activation (Shirakawa et al., 1985).

Over the years, NRF2 has been associated with anti-inflammatory roles in distinct pathologies (Audousset et al., 2021). NRF2 activation attenuated keratinocyte differentiation and inflammation in an imiquimod-induced murine psoriasis model (Ma et al., 2023), ameliorated atherosclerosis by limiting endothelial inflammation and lipid peroxidation (He et al., 2024), promoted bacterial clearance through alveolar macrophages in patients with chronic obstructive pulmonary disease (Harvey et al., 2011), lowered bacterial burden in sepsis (Kong et al., 2011), and decreased inflammation, oxidative stress and apoptosis in colitis (Peng et al., 2023). In a sickle cell disease model, NRF2 activation attenuated lung inflammation and liver damage in mice by eliminating the release of free heme during hemolysis, reducing oxidative stress and expression of pro-inflammatory cytokines in the lungs (Keleku-Lukwete et al., 2018). Additionally, NRF2 activation reduced aortic expression of P-selectin and vascular cell adhesion molecule-1 (VCAM-1), molecules involved in leukocyte recruitment during inflammatory responses (Berlin et al., 1995; Lawrence et al., 1997; Osborn et al., 1989). Indeed, NRF2 activation was found to reduce P-selectin and VCAM-1 promoter activity (Banning and Brigelius-Flohé, 2005) and in endothelial cells, NRF2-dependent suppression of VCAM-1 protected arteries from inflammation (Zakkar et al., 2009).

Corroborating findings from murine and *in vitro* studies, four patients with activating *de novo* mutations in the NRF2-encoding gene *Nfe2l2* developed a multisystem disorder characterized by immunodeficiency, hypohomocysteinaemia, and neurological symptoms (Huppke et al., 2017). These patients suffered from frequent severe lung and skin infections, revealed reduced levels of immunoglobulins, switched memory B cells and were lacking an antibody response to pneumococcal vaccine.

Hence, although increased NRF2 activity may be beneficial under certain inflammatory conditions, its sustained and uncontrolled activation has been shown to contribute to recurrent infections, impaired cellular and humoral immunity, and compromised antimicrobial defense, placing NRF2 at a critical intersection of innate and adaptive immunity. Overall, NRF2 exerts broad immunoregulatory functions, influencing immune cell activation, recruitment, and proliferation, as well as antimicrobial defense and redox homeostasis, in a highly cell-, tissue-, and context-dependent manner.

### 2.3 NRF2 domains control localization and transcriptional activity

Nuclear translocation of NRF2, a Cap ‘n' Collar basic leucine zipper (bZIP) transcription factor (Moi et al., 1994), is regulated by multiple post-translational mechanisms, including phosphorylation by AMP-activated protein kinase and protein kinase Cδ (Joo et al., 2016; Huang et al., 2002), as well as CREB-binding protein (CBP)-dependent acetylation (Kawai et al., 2011). NRF2 contains seven highly conserved NRF2-ECH homology (Neh1-Neh7) domains that confer distinct regulatory functions. The N-terminal Neh2 domain is central to NRF2 turnover and contains the DLG and ETGE motifs required for KEAP1 binding, as well as an α-helix harboring seven lysine residues that serve as sites for KEAP1-mediated polyubiquitination (Itoh et al., 1999; Tong et al., 2006). In addition to the redox-sensitive degron within Neh2, NRF2 stability is regulated by the redox-insensitive Neh6 degron (McMahon et al., 2004), and by retinoid X receptor α-mediated repression through the Neh7 domain (Wang et al., 2013). In contrast, the Neh1, Neh3, Neh4, and Neh5 domains contribute to nuclear localization and transcriptional activation (Silva-Islas and Maldonado, 2018; Nioi et al., 2005; Katoh et al., 2001; Li et al., 2006).

Among these domains, Neh1 is particularly important for transcriptional activity because it contains the bZIP region required for heterodimerization with small musculoaponeurotic fibrosarcoma (sMaf) proteins, including MafK, MafF, and MafG (Motohashi et al., 2004). Heterodimerization with sMaf proteins, together with CBP/p300 lysine acetyltransferase-mediated acetylation of the Neh1 domain, facilitates binding of the NRF2-sMaf complex to AREs within the *cis*-regulatory regions of target genes, thereby inducing the expression of multiple homeostatic and cytoprotective genes (Itoh et al., 1997; Malhotra et al., 2010). Through this canonical transcriptional program, NRF2 regulates a broad network of homeostatic and cytoprotective genes, including genes involved in redox regulation and drug metabolism (Lacher et al., 2015), glutathione biosynthesis (Wild et al., 1998; Chanas et al., 2002), heme metabolism and erythropoiesis (Campbell et al., 2013), as well as glucose metabolism and proteasomal function (Lacher et al., 2015). NRF2 is further shaped by feedback regulation of components of its own signaling pathway. NRF2 induces transcription of its own gene, *Nfe2l2* (Kwak et al., 2002), as well as its negative regulator *Keap1* (Lee et al., 2007), the autophagy receptor *Sqstm1/p62* (Jain et al., 2010), and sMaf proteins (Katsuoka et al., 2005; Malhotra et al., 2010). These feedback mechanisms provide additional control over NRF2 abundance, stability, and transcriptional activity, thereby fine-tuning the magnitude and duration of the NRF2 response.

### 2.4 NRF2 regulates pro-inflammatory gene transcription

Beyond the canonical cytoprotective transcriptional program, NRF2 emerged as an important regulator of innate immune responses. Approximately two decades ago, studies in lipopolysaccharide (LPS)-challenged murine models identified reciprocal regulation between classical NRF2 target genes and inflammatory genes, including the pro-inflammatory cytokines tumor necrosis factor α and interleukin (IL)-6, the chemokines C-C motif chemokine ligand 2 and 10, as well as cyclooxygenase-2 (Nagai et al., 2009; Thimmulappa et al., 2006a; Thimmulappa et al., 2006b). Subsequent studies in murine and human *in vitro* and *in vivo* models have consistently linked NRF2 activation to suppression of inflammatory responses, implicating modulation of NF-κB signaling (Hu et al., 2022; Han et al., 2024), type I interferon (IFN) responses (Gunderstofte et al., 2019; Ryan et al., 2022; Han et al., 2020), and stimulator of IFN genes-dependent signaling (Li et al., 2024; Olagnier et al., 2018). These findings have largely supported an indirect model, where NRF2 limits inflammation through modulation of upstream signaling pathways and cellular redox state.

However, evidence that NRF2 can directly repress inflammatory gene transcription has added an additional layer to this regulatory framework (Kobayashi et al., 2016). Kobayashi et al. demonstrated that NRF2 suppresses LPS-induced transcription of pro-inflammatory cytokines through direct binding to regulatory regions proximal to the *Il1b* and *Il6* promoters, independently of AREs, cellular ROS levels, and NF-κB recruitment. NRF2 binding at these regions directly repressed the recruitment of RNA polymerase II (Pol II), thereby limiting transcription of the respective inflammatory genes. Notably, the NRF2-binding regions proximal to the *Il6* and *Il1b* promoters were found to overlap with binding sites for several pro-inflammatory transcription factors, including the NF-κB subunit RELA (p65), CCAAT/enhancer-binding protein beta (C/EBPβ), and bZIP-transcription factor Jun proto-oncogene. Indeed, Kuhn et al. (2011) reported that macrophage treatment with oxidized low-density lipoprotein increased NRF2 nuclear translocation and transcriptional activity, resulting in increased heme oxygenase expression, attenuated ROS production, decreased C/EBP (β and δ) activity, and inhibited *Il6* and *Il1b* expression upon LPS treatment (Kuhn et al., 2011). Hence, it was proposed that NRF2-mediated repression could involve direct interactions with p65, C/EBPβ, and bZIP-transcription factor Jun proto-oncogene or, alternatively, indirect interactions mediated by MafK association with p65 (Hwang et al., 2013; Kobayashi et al., 2016). However, Kobayashi et al. found that NRF2 activation did not substantially alter recruitment of p65 or C/EBPβ or induce major changes in histone modifications.

Instead, NRF2 activation was associated with constant, rather increased recruitment of CBP and components of the mediator complex. Previous data showed that the mediator subunit MED16 is required for NRF2-driven Pol II phosphorylation and, consequently, for the transcriptional activation of ARE-dependent genes (Sekine et al., 2015). Moreover, these data indicate that in contrast to Liu et al. (2008), who reported that p65 competes with NRF2 for CBP binding and promotes histone deacetylase 3-dependent hypoacetylation at ARE-containing gene loci, repression of pro-inflammatory genes can occur independently of p65-mediated CBP deprivation. Indeed, supporting the findings from Kobayashi et al. (2016), NRF2 activation was reported to deprive p300 from p65, thereby suppressing inflammatory transcription, while increasing NRF2 and p300 association with AREs (Kim et al., 2013). Competition between NRF2 and other transcription factors for limiting pools of shared cofactors, as for example CBP/p300, may add to reduced transcriptional activity, a mechanism often referred to as cofactor squelching (Schmidt et al., 2016). Experimentally, locus-specific occupancy profiling combined with perturbation of candidate cofactors could distinguish altered cofactor availability from direct recruitment of repressive complexes. However, given the increased recruitment of CBP to pro-inflammatory genes upon NRF2 activation, available evidence suggests that cofactor squelching is context-dependent and does not fully account for NRF2-mediated repression of pro-inflammatory genes. Hence, additional coactivators, altered cofactor function, or even recruitment of transcriptional repressors may contribute to a lower pro-inflammatory gene expression upon NRF2 activation. Thus, although NRF2 can directly repress Pol II recruitment to inflammatory genes, the underlying molecular events remain incompletely understood.

NRF2 may also influence inflammatory responses through broader effects on immune-cell differentiation and cytokine production. In murine CD4^+^ T cells, NRF2 activation was shown to suppress T helper 1 differentiation and IFNγ production, while promoting a T helper 2-associated response, characterized by increased secretion of anti-inflammatory IL-4, IL-13, and IL-5 (Rockwell et al., 2012). These findings suggest that the immunoregulatory effects of NRF2 extend beyond direct repression of pro-inflammatory gene transcription and may also involve modulation of broader transcriptional programs governing immune-cell differentiation and cytokine production. Collectively, NRF2 therefore regulates inflammatory responses through multiple, context-dependent mechanisms, ranging from modulation of upstream signaling pathways and cellular redox state to, potentially, direct interactions with transcriptional and chromatin-associated regulators.

### 2.5 NRF2 in MHC class II-dependent antigen presentation

Recent findings linked NRF2 with the control of MHC class II-dependent antigen presentation in both classical antigen-presenting cells (Goess et al., 2021; Rothchild et al., 2019; Rothe et al., 2015; Pham et al., 2026) and primary CAFs (Zheng et al., 2026). Zheng et al. (2026) reported an inverse relationship between NRF2 activity and MHC class II expression in primary CAFs derived from patients with non-small cell lung cancer. NRF2 activation reduced expression of *Ciita* and MHC class II associated genes, whereas NRF2 depletion produced the opposite effect. NRF2 activation was accompanied by increased expression of karyopherin subunit beta 1 (KPNB1), a nuclear transport protein that facilitates shuttling of transcriptional regulators between the cytoplasm and nucleus. Although the authors proposed that KPNB1 may contribute to NRF2-dependent suppression of the MHC class II program, the molecular link between NRF2/KPNB1 and *Ciita* remained unresolved.

Along these lines, recent findings in macrophages provide further evidence for NRF2-dependent regulation of *Ciita*. Using *Mycobacterium tuberculosis* (Mtb)-infected murine AMs, Pham et al. (2026) demonstrated that NRF2 limits the expression of *Ciita*, the master transcriptional regulator of MHC class II gene expression in mice and humans (Masternak et al., 2000; Steimle et al., 1993). Consistent with increased MHC class II surface expression previously observed in NRF2-deficient AMs (Rothchild et al., 2019), Pham et al. provided mechanistic evidence that Mtb-induced NRF2 upregulation suppresses *Ciita*-dependent MHC class II surface expression and, consequently, the capacity of Mtb-infected AMs to activate antigen-specific CD4^+^ T cells. In line with these findings, earlier work by Ma et al. (2006) showed that NRF2 KO enhanced CD4^+^ T cell proliferation, altering CD4^+^/CD8^+^ T cell ratios and multiorgan autoimmune inflammation in mice.

Using murine and human DCs, Rothe et al. (2015) demonstrated that suppressing NRF2 in LPS-treated DCs increased the surface MHC class II expression and the abundance of co-stimulatory molecules, thereby enhancing CD4^+^ T cell differentiation. The authors linked reduced NRF2 activity, resulting from loss of arachidonate 15-lipoxygenase type A and oxidized phospholipids to DC maturation. However, in contrast to the findings made in CAFs and AMs, DC maturation was found to dampen *Ciita* expression (Landmann et al., 2001), suggesting a distinct mechanism by which NRF2 influences MHC class II expression compared to macrophages.

In Mtb-infected AMs, neither Rothchild et al. (2019) nor Pham et al. (2026) detected NRF2 binding at the *Ciita* locus, suggesting that NRF2-mediated repression of *Ciita* occurs indirectly (Pham et al., 2026). This contrasts with the NRF2-dependent repression of pro-inflammatory cytokine genes, such as *Il1b* and *Il6*, for which proximal NRF2 binding-dependent repression of Pol II recruitment was demonstrated (Kobayashi et al., 2016). Thus, the mechanisms underlying NRF2-dependent but NRF2-binding-independent repression of *Ciita* remain unresolved and may differ fundamentally from the direct mechanisms described for pro-inflammatory genes. Additionally, it should be noted that although increased NRF2 activity consistently coincides with reduced *Ciita*/MHC class II expression across these cellular contexts, this apparent common pattern does not necessarily reflect a shared regulatory mechanism.

As the current evidence linking NRF2 to *Ciita* repression remains limited to the findings discussed above, the mechanisms underlying this regulation have not yet been experimentally established. Notably, NRF2-dependent suppression of MHC class II in AMs persisted following innate stimulation and IFNγ exposure, indicating that NRF2 can restrain the antigen-presentation capacity under inflammatory conditions (Pham et al., 2026). These findings suggest that additional downstream or parallel mechanisms may contribute to *Ciita* repression. The following section therefore explores experimentally testable, but currently hypothetical, mechanisms in the following order: (1) recruitment of transcription factors to the *Ciita* locus; (2) redistribution or competition for limiting transcriptional coactivators; (3) altered recruitment of chromatin-remodeling complexes; (4) altered Pol II recruitment or transcriptional initiation; and (5) indirect metabolic or redox effects on the transcriptional machinery.

1. *Ciita* expression is regulated in a cell type- and stimulus-dependent manner by multiple transcriptional regulators, including p65, Sp1 transcription factor, hematopoietic transcription factor PU.1, interferon regulatory factor 8, PR domain zinc finger protein 1 and nuclear factor of activated T cells 5 (Smith et al., 2011; Buxadé et al., 2018). However, whether NRF2 influences *Ciita* expression through any of these transcriptional regulators remains to be investigated.One potential link between NRF2 signaling and *Ciita* repression is provided by KLF transcription factor (KLF) 4. In peritoneal macrophages and RAW 264.7 cells, infection with *Mycobacterium* bovis *Bacillus* Calmette-Guérin induced nitric oxide-dependent recruitment of KLF4 to the *Ciita* promoter, resulting in enhancer of zeste 2 polycomb repressive complex 2 subunit-dependent trimethylation of histone H3 at lysine 27 and subsequent repression of *Ciita* and MHC class II expression (Ghorpade et al., 2013). KLF4 is also a direct transcriptional target of NRF2 (Wang et al., 2025) and is strongly upregulated following Mtb infection (Sahu et al., 2017), raising the possibility that NRF2-dependent induction of KLF4 could contribute to *Ciita* repression. However, a functional connection between NRF2, KLF4, and *Ciita* has not yet been established. Interestingly, KLF4 has also been implicated in the regulation of inflammatory gene expression and was reported to suppress LPS-induced *Il1b* expression in RAW 264.7 macrophages (Liu et al., 2012).
2. Moreover, KLF4 interacts with the transcriptional coactivator and histone acetyltransferases CBP/p300 to regulate target gene expression (Feinberg et al., 2005). Thereby, depending on the specific signaling pathway, KLF4 can either inhibit CPB/p300 recruitment or bind CBP/p300, promoting its own transcriptional activity (Evans et al., 2010; Evans et al., 2007). As discussed earlier, NRF2 interacts with CBP/p300, required for NRF2-dependent transcription through histone acetylation and recruitment/stabilization of the transcriptional machinery (Katoh et al., 2001). Additionally, CBP/p300 is recruited to *Ciita* (Lee et al., 2008; Kwon et al., 2007) and is required for transcriptional initiation of MHC class II genes (Mudhasani and Fontes, 2005; Fontes et al., 1999; Kretsovali et al., 1998; Mudhasani and Fontes, 2002). Whether regulation of *Ciita* expression might be repressed through NRF2-dependent changes in KLF4 expression and redistribution of coactivator CBP/p300 is so far unknown.
3. In addition to the established roles of histone acetylation in NRF2-mediated activation of antioxidant genes and in *Ciita* transcription, changes in chromatin accessibility resulting from altered recruitment of chromatin-remodeling complexes may act cooperatively. Indeed, INFγ-mediated induction of *Ciita* expression depends on SWI/SNF-related BAF chromatin-remodeling complex subunit ATPase 4 (BRG1)-binding to distal enhancers, allowing p300 recruitment and histone modifications (Ni et al., 2008). Additionally, Mtb infection has been shown to suppress IFNγ-dependent BRG1 recruitment to *Ciita* promoter IV, accompanied by reduced local histone acetylation and consequently impaired *Ciita* and MHC class II expression (Pennini et al., 2006). On the other side, NRF2 recruits BRG1 to regulatory regions of antioxidant genes, which in turn is required for Pol II recruitment (Zhang et al., 2006). Additionally, another subunit of the SWI/SNF-related BAF chromatin-remodeling complex, double PHD fingers 2, is required for the NRF2-mediated transcriptional response. In macrophages, loss of PHD fingers 2 prevented BRG1-recruitment to NRF2-controlled enhancers, impairing both antioxidant and anti-inflammatory, while enriching IFNγ response genes (Mas et al., 2023). Given the reported interaction between NRF2 and BRG1, altered BRG1 recruitment or activity could therefore represent a potential link between NRF2 signaling and chromatin-dependent regulation of *Ciita.* Whether NRF2 influences BRG1-dependent regulation of *Ciita* remains to be uncovered.
4. In contrast to pro-inflammatory genes, where NRF2 binding to proximal regulatory regions was shown to directly suppress Pol II recruitment and thereby transcription (Kobayashi et al., 2016), NRF2-dependent regulation of Pol II recruitment at the *Ciita* locus remains hypothetical. Unlike the downstream MHC class II genes, for which recruitment and activation of Pol II have been extensively characterized (Spilianakis et al., 2003), the mechanisms governing recruitment and activation of the basal transcriptional machinery at the *Ciita* locus remain comparatively poorly defined.
5. Finally, NRF2-dependent metabolic and redox remodeling could indirectly influence the transcriptional machinery controlling *Ciita* (Ryan et al., 2022). NRF2-dependent alterations in lipid and carbohydrate metabolism affect the availability of metabolites serving as substrates or cofactors for chromatin-modifying enzymes and thereby influence transcriptional states. However, whether such metabolic or redox changes contribute to NRF2-dependent *Ciita* repression has not been investigated. Moreover, NRF2 can inhibit transcription independently of changes in cellular ROS (Kobayashi et al., 2016), indicating that redox-mediated effects are not required for NRF2-dependent transcriptional repression.

Collectively, the available evidence indicates that NRF2 can modulate both antigen-presentation and inflammatory gene programs through shared as well as context-dependent mechanisms (Figure 1). Although mechanistic studies of NRF2-mediated regulation of inflammatory genes have identified several potential mechanisms of interaction with the transcriptional machinery, a coherent molecular mechanism has not been established, neither for inflammatory gene regulation nor for NRF2-dependent *Ciita* repression. Notably, the absence of NRF2 binding at the *Ciita* locus contrasts with the direct NRF2 occupancy reported at several inflammatory gene loci, suggesting that *Ciita* may be regulated through a mechanistically distinct, likely indirect pathway. For *Ciita*, factors such as BRG1, KLF4, and CBP/p300 represent plausible molecular links based on their established roles in transcriptional or chromatin regulation, but their involvement in NRF2-dependent *Ciita* repression remains untested. Thus, the mechanisms proposed here are considered experimentally testable rather than established pathways.

**FIGURE 1:**
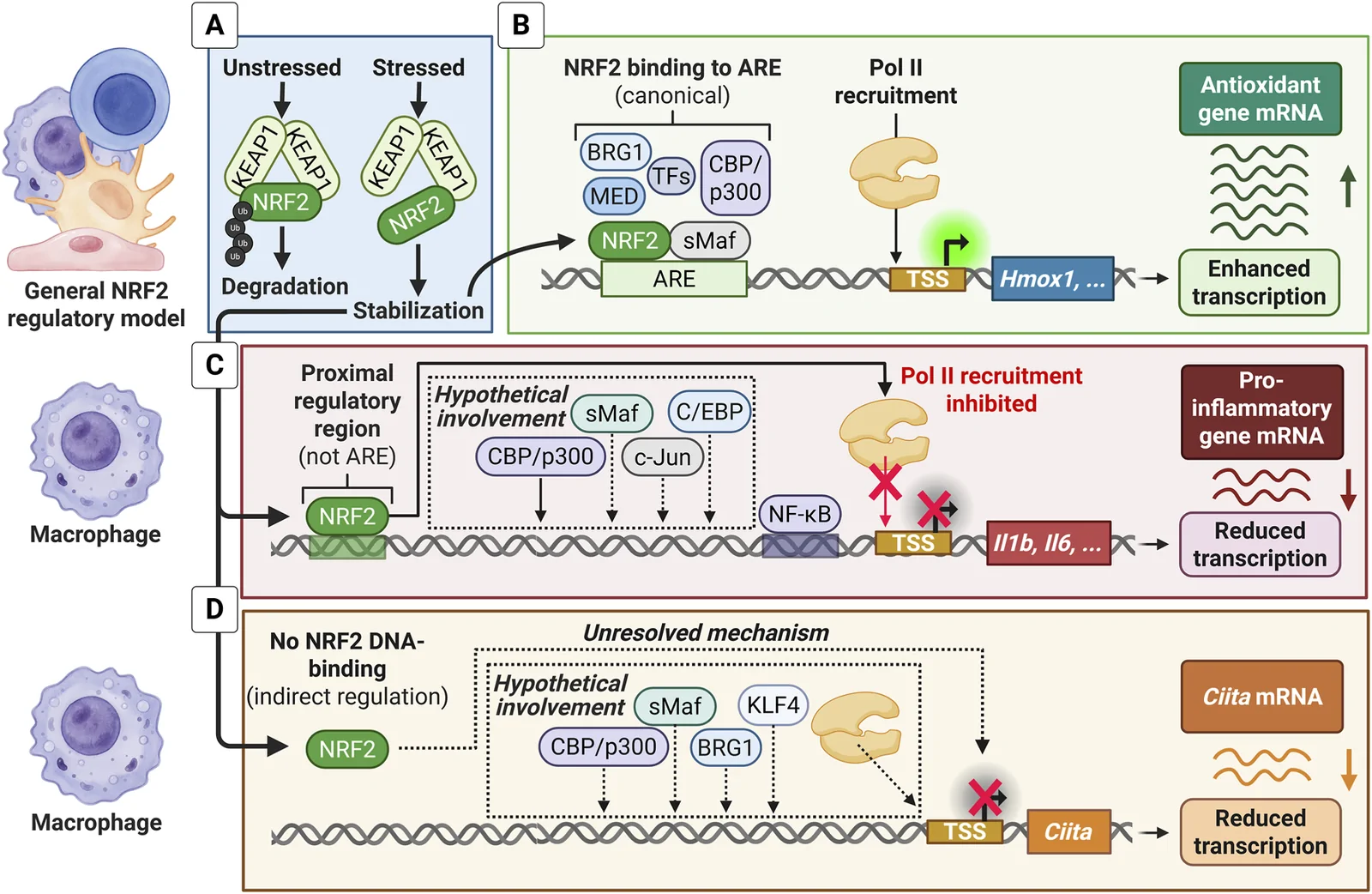
NRF2 transcriptional regulation of antioxidant and pro-inflammatory genes as well as the MHC class II transactivator *Ciita*. **(A)** Canonical NRF2 activation is initiated by oxidative (reactive oxygen species, ROS) or electrophilic modification of cysteine residues within KEAP1, the negative regulator and cytoplasmic binding partner of NRF2. Upon NRF2 stabilization through decreased KEAP1-CUL3-mediated NRF2 degradation, NRF2 translocates to the nucleus, where it regulates the transcription of antioxidant **(B)** and immunoregulatory genes **(C,D)**. **(B)** Following CREB-binding protein (CBP)/p300 lysine acetyltransferase-mediated acetylation, NRF2 binds antioxidant response elements (AREs) through heterodimerization with small musculoaponeurotic fibrosarcoma (sMaf) proteins. Interactions with additional transcription factors (TFs) can further enhance NRF2-dependent transcription. Chromatin remodeling by the SWI/SNF-related BAF chromatin-remodeling complex ATPase 4 (BRG1), histone acetylation by CBP/p300, and recruitment of the mediator complex (MED) by NRF2 facilitate RNA polymerase II (Pol II) recruitment and phosphorylation. Consequently, transcription of antioxidant genes, including heme oxygenase 1 (*Hmox1*), is enhanced at their promoters/transcription start sites (TSS). **(C)** NRF2-mediated repression of pro-inflammatory genes has been investigated in lipopolysaccharide-treated murine macrophages. NRF2 binding to non-ARE regions in proximity to the *Il1b* and *Il6* promoters impairs Pol II recruitment, while maintaining nuclear factor kappa B (NF-κB) binding. Although these mechanisms have not been investigated and remain hypothetical, canonical NRF2 binding to sMaf proteins, dependence on CBP/p300, and interactions with transcription factors such as CCAAT/enhancer-binding protein (C/EBP) and Jun proto-oncogene (c-Jun) may contribute to this regulatory process. The mechanisms underlying NRF2-binding-dependent suppression of Pol II recruitment at pro-inflammatory genes remain unresolved. **(D)** In addition to lowered surface expression of major histocompatibility complex (MHC) class II molecules in dendritic cells, recent findings in alveolar macrophages have identified NRF2-driven transcriptional repression of the MHC class II transactivator (*CIITA*). In contrast to canonical NRF2 target genes and pro-inflammatory genes, direct DNA binding by NRF2 does not appear to be required for repression of *Ciita* expression. Based on the available literature, experimentally testable candidates in the NRF2-dependent *Ciita* regulation may include the interaction of NRF2 with canonical co-activators, chromatin-remodeling, histone-modifying enzymes, and regulation of transcription factors such as KLF transcription factor (KLF) 4. Moreover, to date, the role of Pol II recruitment in NRF2-mediated repression of *Ciita* has not been examined.

Given that monocytes from patients with active SLE exhibited reduced predicted NRF2 activity, alongside decreased frequencies of MHC class II-positive monocytes, the relationship between NRF2 activity and MHC class II expression appears to be highly dependent on the cellular, inflammatory, and disease context. Thus, although NRF2 can restrain excessive inflammatory responses and limit tissue damage under certain conditions, reduced NRF2 activity may enhance immune cell activation and, in some settings, contribute to chronic inflammation, autoimmunity, or organ damage. Conversely, excessive or sustained NRF2 activity may dampen immune responses that are required for effective host defense. These context-dependent effects highlight potential immunological trade-offs that should be considered when evaluating NRF2-targeting therapeutics.

## 3 Conclusion

To date, numerous *in vivo* and *in vitro* studies have established that NRF2 induces antioxidant and stress-response genes, while concomitantly repressing pro-inflammatory gene expression. Although alterations in MHC class II expression following NRF2 depletion have been reported for nearly a decade, only recently has NRF2-dependent transcriptional regulation of the MHC class II transactivator been uncovered. Like ARE-mediated gene regulation, effects of NRF2 on pro-inflammatory genes and *Ciita* may involve additional NRF2-associated transcription factors, chromatin remodeling enzymes, histone deacetylases, or methyltransferases. In addition to the recruitment of transcriptional repressors, cofactor squelching, or competition among transcription factors for a limited pool of shared transcriptional coactivators may contribute to reduced transcriptional activity. Mechanistic studies of NRF2-mediated regulation of inflammatory genes have identified several potential points of interaction with the transcriptional machinery, but a coherent molecular mechanism for NRF2-dependent transcriptional repression has not been established.

Notably, NRF2 binding was not detected at the *Ciita* locus, in contrast to direct NRF2 occupancy reported at several inflammatory gene loci, supporting the notion that *Ciita* is regulated through an indirect and potentially mechanistically distinct pathway. Unlike the direct NRF2 occupancy reported at several pro-inflammatory gene loci, *Ciita* may be subject to a mechanistically distinct, indirect mode of NRF2-dependent repression. As observed for pro-inflammatory gene regulation, a broad range of transcription factors, cofactors, and chromatin-modifying enzymes may therefore contribute to the regulation of *Ciita*. Factors such as BRG1, KLF4, CBP/p300, and other transcriptional or chromatin regulators represent plausible candidates for interaction, but their contribution to NRF2-dependent *Ciita* repression remains untested. Future research should investigate whether NRF2 alters BRG1 occupancy at *Ciita* promoters, whether candidate cofactors are redistributed following NRF2 activation, and whether Pol II recruitment at *Ciita* changes in an NRF2-dependent manner. Importantly, these mechanisms may operate independently of direct NRF2 DNA-binding.

The functional consequences of this regulatory axis may be particularly relevant in settings in which inflammatory responses and antigen presentation must be balanced. Given the role of NRF2 in immune-cell activation, recruitment, and proliferation during both inflammatory and homeostatic conditions, further attention to the transcriptional mechanisms affecting cytokine and *Ciita* expression is warranted, as both inflammatory signaling and MHC class II-dependent antigen presentation are fundamental to pathogen recognition and host defense. Therefore, pharmacological NRF2 activation may be beneficial under chronic inflammatory or autoimmune settings, while NRF2-dependent repression of *Ciita* and MHC class II expression may potentially limit antigen presentation during infections or conditions in which effective MHC class II-dependent immune responses are required. Understanding molecular mechanisms and context dependent NRF2-mediated regulation of both, inflammatory genes and *Ciita* will therefore be important for predicting the immunological consequences of therapeutic NRF2 modulation.

## Glossary

AM: Alveolar macrophage
ARE: Antioxidant response element
bZIP: Basic leucine zipper
CAFs: Cancer-associated fibroblasts
C/EBP: CCAAT/enhancer-binding protein
CBP: CREB-binding protein
CCL: C-C Motif Chemokine Ligand
CNC: Cap ‘n' Collar
CUL3: Cullin 3
DC: Dendritic cell
FcεRIγ: Fc receptor, IgE, high-affinity I, γ-polypeptide
IFN: Interferon
IKKβ: Nuclear factor kappa B kinase subunit beta
IL: Interleukin
KEAP1: Kelch-like ECH-associated protein 1
KLF: KLF transcription factor
KO: Knockout
KPNB1: Karyopherin subunit beta 1
LPS: Lipopolysaccharide
MHC: Major histocompatibility complex
Mtb: *Mycobacterium tuberculosis*
Neh: NRF2-ECH homology domain
NF-κB: Nuclear factor kappa B
NRF2: NFE2-like BZIP transcription factor
p300: EP300 lysine acetyltransferase
p65: NF-κB subunit RELA
Pol II: RNA polymerase II
ROS: Reactive oxygen species
SLE: Systemic lupus erythematosus
BRG1: SWI/SNF-related BAF chromatin-remodeling complex subunit ATPase 4
SQSTM1/p62: Sequestosome 1
VCAM-1: Vascular cell adhesion molecule-1
